# Barriers and facilitators for GPs in dementia advance care planning: A systematic integrative review

**DOI:** 10.1371/journal.pone.0198535

**Published:** 2018-06-20

**Authors:** Bram Tilburgs, Myrra Vernooij-Dassen, Raymond Koopmans, Hans van Gennip, Yvonne Engels, Marieke Perry

**Affiliations:** 1 Department of IQ Healthcare, Radboudumc, Nijmegen, The Netherlands; 2 Department of Primary and Community Care, Radboudumc, Nijmegen, The Netherlands; 3 Radboudumc Alzheimer Centre, Nijmegen, The Netherlands; 4 Joachim and Anna, Centre for Specialized Geriatric Care, Nijmegen, The Netherlands; 5 Independent Educational Researcher, Family carer, Nijmegen, The Netherlands; 6 Department of Anaesthesiology, Pain and Palliative Medicine, Radboudumc, Nijmegen, The Netherlands; 7 Department of Geriatric Medicine, Radboudumc, Nijmegen, The Netherlands; University of Western Australia, AUSTRALIA

## Abstract

**Background:**

Due to the disease’s progressive nature, advance care planning (ACP) is recommended for people with early stage dementia. General practitioners (GPs) should initiate ACP because of their longstanding relationships with their patients and their early involvement with the disease, however ACP is seldom applied.

**Aim:**

To determine the barriers and facilitators faced by GPs related to ACP with people with dementia.

**Data sources:**

We systematically searched the relevant databases for papers published between January 1995 and December 2016, using the terms: primary healthcare, GP, dementia, and ACP. We conducted a systematic integrative review following Whittemore and Knafl’s method. Papers containing empirical data about GP barriers and/or facilitators regarding ACP for people with dementia were included. We evaluated quality using the Mixed-Method-Appraisal-Tool and analyzed data using qualitative content analysis.

**Results:**

Ten qualitative, five quantitative, and one mixed-method paper revealed four themes: timely initiation of ACP, stakeholder engagement, important aspects of ACP the conversation, and prerequisites for ACP. Important barriers were: uncertainty about the timing of ACP, how to plan for an uncertain future, lack of knowledge about dementia, difficulties assessing people with dementia’s decisional capacities, and changing preferences. Facilitators for ACP were: an early start when cognitive decline is still mild, inclusion of all stakeholders, and discussing social and medical issues aimed at maintaining normal life.

**Conclusion:**

Discussing future care is difficult due to uncertainties about the future and the decisional capacities of people with dementia. Based on the facilitators, we recommend that GPs use a timely and goal-oriented approach and involve all stakeholders. ACP discussions should focus on the ability of people with dementia to maintain normal daily function as well as on their quality of life, instead of end-of-life-discussions only. GPs need training to acquire knowledge and skills to timely initiate collaborative ACP discussions.

## Introduction

Dementia is a progressive and chronic condition with a median survival of 7 to 10 years [1]. Worldwide, 50 million people suffer from dementia and this number is expected to increase to 152 million by 2050 [2]. During the disease process, people with dementia undergo a declining cognitive capacity resulting in an increased dependency on others [3]. It is estimated that in the USA and Europe, approximately 6% of the population aged over 60, and 45% aged over 90, have dementia [3]. Above the age of 65, 10% of all deaths in men and 15% of all deaths in woman can be attributed to dementia [3]. In addition, data from UK GP practices shows that 19% of people with dementia more commonly had five or more additional physical conditions than those without dementia (13,4%) [4].

Dementia care should be proactive, patient-centered, and focus on improving quality of life (QoL) and daily functioning [5–7]. To accomplish this, advance care planning (ACP) is recommended [7, 8]. ACP can be defined as ‘a timely and cyclic assessment of future health issues by discussions between patients, their family and healthcare professionals, taking wishes and preferences for future care into account’ [9–11]. During ACP, medical, psychological, social and existential subjects can be addressed, and people are given the opportunity to discuss what they do and do not want regarding their future care [12]. ACP may then result in the documentation of preferences for future care. Advance directives, decisions to refuse treatment, living wills and/or lasting power of attorney, are structured examples of this [10]. Worthy of note is that most studies on the effectiveness of ACP primarily addressed medical, end-of-life related topics, which neither reflects the heterogeneity of the disease nor the broad definition advised [11].

ACP has been shown to improve the concordance between healthcare preferences and care delivered in different adult populations [13]. It appears to increase the completion of advance directives, to enhance communication between patients, family carers and healthcare professionals, and to stimulate conversations about future wishes and preferences [13, 14]. By registering these preferences the frail elderly undergo less aggressive treatment, less admittance to hospital, less anxiety, stress and depression, and increased death in a trusted environment [15]. For people with dementia living in nursing homes, ACP reduces both hospital admissions and healthcare costs [16]. However, because of the more common occurrence of advanced dementia in nursing homes, residents are often deemed less capable of making their own decisions and are therefore unlikely to be invited to actively participate in ACP [16]. In contrast, most people with dementia who live at home have mild to moderate dementia [17] and therefore are able to express their preferences [18, 19].

Most home-dwelling people with dementia receive care from a general practitioner (GP). Because of GPs’ longstanding relationships with their patients, they are the professionals most suited to initiate ACP in this group [20]. Research, however, has indicated that of the non-cancer patients who had non-sudden deaths, only 24% had an ACP conversation with their GP, and only 5.3% had a written plan [21]. In addition, dementia is negatively associated with discussing treatment preferences, which indicates that ACP within dementia has its own specific challenges [22].

In order to gain a better understanding of these challenges, in this integrative review of the literature, we reviewed barriers and facilitators to the initiation of ACP by GPs for people with dementia.

## Methods

We used the integrative review methodology described by Whittemore and Knafl [23]. In contrast to traditional systematic reviews, this method allows the simultaneous inclusion of theoretical, quantitative, and qualitative studies. By systematically searching, evaluating, and analyzing relevant studies with different methodologies, were able to better integrate and understand all aspects related to our research question [23].

After having determined our research aim, we searched Embase, Psychinfo, Medline, Cinahl and the Cochrane Library databases using a combination of the following search terms: primary healthcare, general practitioner, dementia, and advance care planning as MeSH terms, free text words, and equivalent index terms (Table 1). The search was limited to English language peer reviewed journals published between January 1, 1995 and December 31, 2016. We chose 1995 as a starting point as literature on ACP in primary care prior to 1995 is scarce [24]. Additionally, we hand-searched the reference lists of relevant studies.

**Table 1:** Search strategies for Medline, Psychinfo, CINAHL.

Medline	Psychinfo	CINAHL
(exp Primary Health Care/ OR exp General Practitioners/ OR exp Community Health Services/ OR ((primary adj3 care) OR (health adj3 care adj3 primary) OR (primary adj3 health adj3 care)).ti,kw,ab. OR (general adj3 practitioner?).ti,kw,ab. OR (community adj3 health adj services).ti,kw,ab. OR (family adj3 medicine).ti,kw,ab. OR exp Physicians, Family/ OR (physician? adj3 family).ab,kw,ti. OR (family adj 3 physician?).ab,ti,kw. OR (family adj3 doctor?).ab,kw,ti. OR (primary adj3 physician?).ti,ab,kw. OR (community adj3 health adj3 care).ti,ab,kw.) AND (exp Advance Care Planning/ OR exp Advance Directives/ OR (advance adj3 care adj3 planning).ti,kw,ab. OR ACP.ti,kw,ab. OR (advance adj3 medical adj3 plan?).ti,kw,ab. OR (advance adj3 health adj3 care adj3 plan?).ti,kw,ab. OR (advance adj3 healthcare adj3 plan?).ti,kw,ab. OR (advance adj3 health-care adj3 plan?).ti,kw,ab. OR (advance adj3 directive?).ti,kw,ab. OR (advance adj3 medical adj3 directive?).ti,kw,ab. OR (end adj3 life adj3 communicat*).ti,kw,ab. OR (end-of-life adj3 communicat*).ti,ab,kw. OR (life adj3 sustaining adj3 treat* adj3 preference?).ti,kw,ab. OR (life-sustaining adj3 treatment adj3 preference?).ti,kw,ab. OR (end adj3 life adj3 decision adj3 making).ti,kw,ab. OR (end-of-life adj3 decision adj3 making).ti,kw,ab. OR (living adj3 will?).ti,kw,ab. OR exp patient participation/ OR(patient adj3 participation).ti,kw,ab. OR (patient adj3 involvement).ti,kw,ab. OR (advance adj3 decision adj3 making).ti,kw,ab. OR (advance adj3 decision?).ti,kw,ab. OR (shared adj3 decision adj3 making).ti,kw,ab. OR exp Life support Care/ OR (life adj3 suppORt adj3 care).ti,kw,ab. OR (end adj3 life adj3 decision?).ti,ab,kw.) AND (exp Dementia/ OR (alzheimer* adj3 diseas*).ti,kw,ab. OR dement*.ti,kw,ab.)	(exp Primary Health Care/ OR exp General Practitioners/ OR ((primary adj3 care) or (health adj3 care adj3 primary) or (primary adj3 health adj3 care)).ti,id,ab. OR (general adj3 practitioner?).ti,id,ab OR (community adj3 health adj services).ti,id,ab OR (family adj3 medicine).ti,id,ab. OR exp Family Physicians/ OR (family adj3 physician?).ti,ab,id. OR (community adj health adj care).ti,id,ab. OR (family adj3 doctor?).ti,ab,id. OR (primary adj3 physician?).ab,ti,id.) AND (exp Advance Directives/ OR (advance adj3 care adj3 planning).ti,id,ab. OR ACP.ti,id,ab. OR (advance adj3 medical adj3 plan?).ti,id,ab. OR (advance adj3 health adj3 care adj3 plan?).ti,id,ab. OR (advance adj3 healthcare adj3 plan?).ti,id,ab. OR (advance adj3 health-care adj3 plan?).ti,id,ab. OR (advance adj3 directive?).ti,id,ab. OR (advance adj3 medical adj3 directive?).ti,id,ab. OR (end adj3 life adj3 communicat*).ti,id,ab. OR (end-of-life adj3 communicat*).ti,ab,id. OR (life adj3 sustaining adj3 treat* adj3 preference?).ti,id,ab OR (life-sustaining adj3 treatment adj3 preference?).ti,id,ab OR (end adj3 life adj3 decision adj3 making).ti,id,ab. OR (end-of-life adj3 decision adj3 making).ti,id,ab. OR (living adj3 will?).ti,id,ab. OR exp Client Participation/ OR (client adj3 participation).ti,id,ab. OR (patient adj3 participation).ti,id,ab. OR (client adj3 involvement).ti,id,ab. OR (patient adj3 involvement).ti,id,ab. OR (advance adj3 decision adj3 making).ti,id,ab. OR (advance adj3 decision?).ti,id,ab. OR (shared adj3 decision adj3 making).ti,id,ab. OR exp Palliative Care/ OR (palliative adj3 care).ti,id,ab. OR exp Life Sustaining Treatment/ OR (life adj3 sustaining adj3 treat*).ti,id,ab.) AND (exp Dementia/ OR (alzheimer* adj3 diseas*).ti,id,ab. OR dement*.ti,id,ab. OR exp Alzheimer’s Disease/)	(TI primary physician OR AB primary physician OR TI community health OR AB community health OR (MH "Community Health Services+") OR TI family doctor OR AB family doctor OR TI family medicine OR AB family medicine OR TI primary health care OR AB primary health care OR TI primary healthcare OR AB primary healthcare OR (MH "Primary Health Care") OR TI general practitioner OR AB general practitioner OR (MH "Physicians, Family") OR TI family physician OR AB family physician) AND ((MH "Dementia+") OR ((TI dementia) OR (AB dementia)) OR ((TI alzheimer’s disease) OR (AB alzheimer’s disease))) AND (TI end of life decisions OR AB end of life decisions OR TI life sustaining treatment preferences OR AB life sustaining treatment preferences OR TI palliative care OR AB palliative care OR (MH "Palliative Care") OR TI end of life decision making OR AB end of life decision making OR TI shared decision making OR AB shared decision making OR TI advance decision OR AB advance decision OR TI patient involvement OR AB patient involvement OR TI patient participation OR AB patient participation OR TI living will OR AB living will OR TI end of life decisions OR AB end of life decisions OR TI life sustaining treatment OR AB life sustaining treatment OR TI end of life communication OR AB end of life communication OR (MH "Decision Making, Patient+") OR (MH "Decision Making, Family") OR TI advance medical directives OR AB advance medical directives OR TI advance health directive OR AB advance health directive OR (MH "Advance Care Planning") OR ((TI advance care planning) OR (AB advance care planning)) OR (MH "Advance Directives+") OR ((TI advance directives) OR (AB advance directives)))

**Fig 1:**
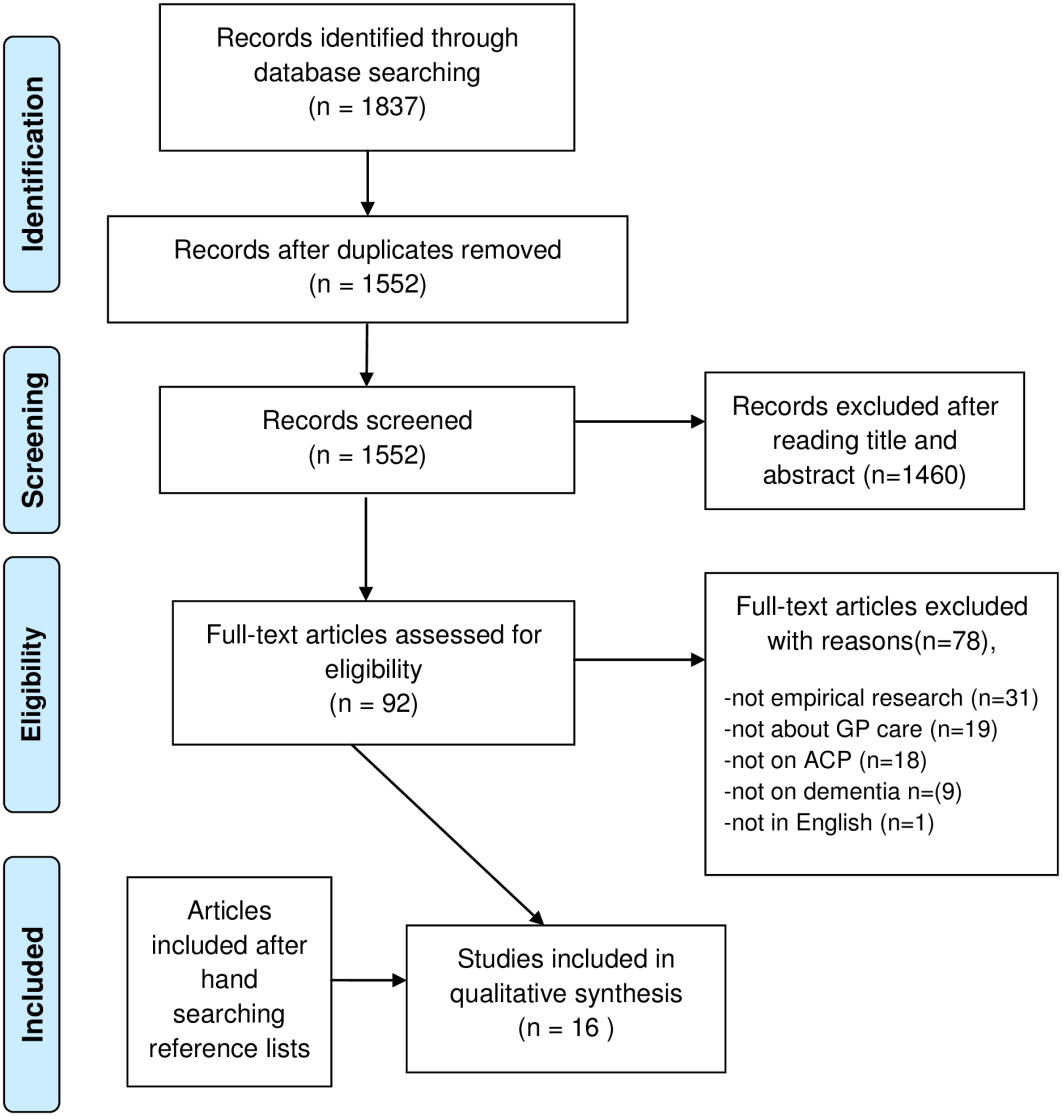
Preferred Reporting Items for Systematic Reviews and Meta-analyses (PRISMA) flow diagram.

We followed the Preferred Reporting Items for Systematic Reviews and Meta-Analyses (PRISMA) guidelines for article selection [25, 26]. Empirical papers containing quantitative and/or qualitative data about barriers and/or facilitators for ACP with people with dementia by GPs were included. After removing duplicate articles, three researchers (BT, AS, VH) independently excluded papers after reading the title and abstract. In a few cases, the title obviously showed that the paper did not address our research aim. Then the abstract was not read. The remaining articles were then read, full-text. Articles were excluded if they did not describe empirical research, were not about dementia, ACP, general practice, or were not written in English (Fig 1). After each step, we compared results and discussed any difference. In cases of disagreement, two other researchers (MP, YE) were consulted.

To evaluate the data, we determined the methodological quality of the studies. Two researchers (BT, SK) independently used the Mixed Methods Appraisal Tool (MMAT), a tool designed for the appraisal of complex systematic reviews that include qualitative, quantitative and mixed methods studies [27]. The MMAT consists of two screening questions for five different kinds of methodological research (qualitative research, randomized controlled quantitative research, non-randomized controlled quantitative research, observational descriptive quantitative research and mixed methods research). These questions address the clarity of the research question and whether the data collected are sufficient to answer the research questions. In addition, the MMAT consists of five specific sets with four quality criteria for each type of research. Each type of research is thus judged within its own methodological domain. Ratings vary between 0% (no quality criteria met) and 100% (all four quality criteria met)[27, 28].

We analyzed the data, aiming for a thorough interpretation of primary sources and synthesis of evidence [23]. Since in qualitative research the emphasis is on exploration and classification and quantitative research focuses on enumeration, integration of data is complicated. Qualitative and quantitative results were therefore analyzed separately using qualitative content analysis. Thereto, the results sections of all the papers were transferred to ATLAS.ti. version 7. Using this software all passages in the result sections on ACP facilitators and barriers were given conceptual labels representing their underlying content. This coding process was performed independently by three researchers (BT, AW, SK), followed by several group sessions where researchers (YE, MP, MVD, HvG, BT) merged codes with similar meanings and categorized them. Using an affinity diagram, we combined these categories into themes representing the underlying codes and categories [29–31]. The merged codes, categories and themes of all qualitative and quantitative studies were tabled, (Tables 2 and 3) enabling data comparison, interpretation and integration [23].

**Table 2:** Themes, categories and codes of the included qualitative articles.

Themes	Categories	Codes	
		Facilitators	Barriers
*A timely initiation of ACP*	*The start of ACP*	A timely start facilitates ACP [32, 34–36] (P,C,HP)	The right timing for ACP is difficult to determine[34, 37, 38] (P,C,HP)
		The dementia diagnosis stimulates patients to think about the future[32, 35, 36] (P,C)	The patients denial/resistance of the dementia diagnosis hinders ACP[35, 36](C)
		Because of the cognitive decline, when ACP starts early more participation is possible[32, 34, 35, 37, 38] (C,HP)	The denial of future problems hinders ACP [35]
		ACP gives patients time to think about the future[32] (P)	
	*Initiating ACP*	High impact events can prompt ACP[35] (C)	ACP is not initiated because it might cause stress or fear with the patient[34, 37] (C,HP)
		The GP should take the initiative for ACP[32, 37] (C,HP)	It is not always clear who should take the initiative for ACP[32, 37, 38] (HP)
		ACP stimulates discussions about the future[32] (C)	
*Stakeholders engagement*	*Relations between stakeholders*	A good relationship between the patient/family and the GP facilitates ACP[32] (HP)	Carers find that the difficult relationship between them and the patients hinders ACP[36] (C)
	*Involving all stakeholders in ACP*	If the patient is no longer capable of making decisions, others will[34, 36] (P,C)	The unawareness of the dementia diagnosis hinders ACP [37] (HP)
		It is preferred to carry out ACP with all stakeholders[34, 36, 44, 45] (P,C)	The stakeholders assessment of the patients decisional capacity is limited in consisted and hinders ACP[38] (C)
			Carers find taking the responsibility for ACP decisions difficult [36] (C).
*Important aspects of the ACP conversation*	*Informing the patient*	Providing realistic information increases empowerment[47] (C)	Patients and carers are insufficiently informed about the diagnosis, disease trajectory, care and treatment options[32–34, 36, 37] (P,C,HP)
			Patients and carers lack knowledge about the purpose of ACP or are unaware of the existence [34, 45] (P,C)
			GPs provide information selectively because they feel patient/carers cannot cope[47] (C)
	*Exploring the patient’s wishes and needs*	The GP must ask for the patients needs directly[37] (C)	The limitations of healthcare can be a barrier for ACP[34, 36] (P,C)
			The costs of legal matters are high and limit ACP[38] (HP
	*Decision making in ACP*	The patients pursuit for a normal level of function influences ACP decision making [44] (P)	
		Burdensome interventions take place when preferences are unknown[33] (C,HP)	
		The carers previous experiences with other dementia patients influences ACP[36] (C)	
		Financial matters and the power of attorney must be a topic in ACP[34–36, 38] (P,C,HP)	
		Stakeholders prefer informal ACP discussions [34, 37] (P,C)	
		The preservation of QOL influences ACP decisions [36] (C)	
		The use of decision aids can support ACP decision making[32, 47] (P,C)	
	*Documentation of ACP*	Documenting ACP makes patient wishes available to all stakeholders[32, 34, 35, 38] (P,C,HP)	ACP is not documented because future wishes/circumstances might change[34, 37]
		Trough ACP wishes are known by all stakeholders[32] (P,C)	ACP decisions are not documented because of feelings of guilt/disloyalty[34] (C)
*Prerequisites of ACP*	*Abilities of the GP regarding ACP*	GP’s knowledge about the diagnosis, disease trajectory, care and treatment options facilitate ACP[37] (HP)	GP’s lack knowledge about the legal status of ACP[37, 38] (HP)
		Training the GP is essential for ACP [32] (HP)	
		Good communication skills of the GP facilitate ACP [32, 37] (HP)	
	*Stakeholders attitudes towards ACP*	ACP provides self protection, feelings of relief and takes away concerns about the future [32] (P)	Discussing the future can be dispiriting[32] (P)
		ACP must be a cyclical process so decisions are regularly reviewed[34] (C)	There are doubts about the added value of ACP [44] (C)
		Previous experiences facilitate ACP [37] (HP)	The patients personality can hinder ACP[35] (C)
			ACP is not possible because patients preferences might change[33, 37, 38] (C, HP)
			ACP is difficult because the future is unpredictable [32, 34, 38, 45] (P,C,HP)
			Doubts if the decisions made in ACP are feasible [34, 38, 45] (P,C,HP)
			Patients/carers are not oriented on the future[32, 34–36, 45] (P,C)
			Stakeholders have doubts about the added value of ACP[38](HP)
			A negative attitude towards ACP is a barrier for having these discussions[37] (HP)
			Some stakeholders feel that ACP is outside their professional remit[38] (HP)
	*The continuous process of ACP*	ACP must be a cyclical process so decisions are regularly reviewed [32, 34, 44] (P)	

P: stated by the patient; C: stated by the carer; HP: stated by the GP/healthcare professional

**Table 3:** Themes, categories and codes of the quantitative articles.

Themes	categories	Codes	
		Facilitators	Barriers
*A timely initiation of ACP*	*The start of ACP*	Because of the cognitive decline, when ACP starts early more participation is possible[41–43] (P,HP)	The right timing of ACP is difficult to determine [39, 40] (HP)
		A timely start facilitates ACP[39] (HP)	PWD’s denial/resistance of the dementia diagnosis hinders ACP[39, 43] (HP)
	*Initiating ACP*	The GP should take the initiative for ACP[39, 40] (HP)	ACP is not initiated because it might cause stress or fear with PWD [39] (HP)
*Stakeholder engagement*	*Relations between stakeholders*		
	*Involving all stakeholders in ACP*	It is preferred to carry out ACP with all stakeholders[39, 46] (P,HP)	PWD are only limitary involved in ACP [41] (P)
		PWD’s participation is possible in all phases of dementia[41, 46] (P)	The assessment of the PWD’s decisional capacity is limited, inconsistent and hinders ACP[41] (C,HP)
		PWD are able to participate about values longer[46] (P)	
*Key aspects of the ACP conversation*	*Informing the patient*	PWD should be informed about the diagnosis, disease trajectory, care and treatment options [39] (HP)	
	*Exploring the patient’s wishes and needs*	PWD’s preferences for ACP depend on the domain of the topic discussed[41] (P)	
	*Decision making in ACP*	An advance directive is important in dementia [39] (HP)	
	*Documentation of ACP*		
*Prerequisites of ACP*	*Abilities of the GP regarding ACP*		
	*Stakeholders attitudes towards ACP*	A positive attitude towards ACP is a facilitator for having these discussions[43] (P)	
	*The continuous process of ACP*		

P: stated by the patient; C: stated by the carer; HP: stated by the GP/healthcare professional

## Results

We selected 16 papers (Tables 4 and 5) published after 2004; most research was conducted in the UK (N = 7) followed by the USA (N = 4). Study populations consisted of people with dementia, family carers or GPs, sometimes in combination with other healthcare professionals. Ten were qualitative studies and five were quantitative studies with cross-sectional designs; one paper described an explorative mixed method study.

**Table 4:** Description of the selected qualitative studies.

Author	Design	Participants and settings	Main findings	Themes	MMAT*
Lawrence et al. United Kingdom, 2011	A qualitative design using in-depth interviews with healthcare professionals and family carers	27 bereaved FCs and 23 healthcare professionals from the community, care homes, general hospitals and continuing care units	The timing was considered crucial. ACP should not start too soon because this would cause distress and not to late because of the cognitive decline. PWD and FCs felt insufficiently informed about dementia and ACP. No one felt the responsibility to start ACP.	Timely initiation of ACP, Important aspects of the ACP conversation Prerequisites of ACP	50% of the criteria met
De Vleminck et al. Belgium, 2014	A qualitative exploratory design using focus group interviews	36 GPs from local peer-review groups	The lack of familiarity with the terminal phase of dementia, the lack of key moments to initiate ACP, the patients lack of awareness of their diagnosis and prognosis and the fact that patients do not initiate ACP themselves are barriers to conducting ACP. Familiarity with palliative care was considered a facilitator	Timely initiation of ACP Stakeholder engagement, Important aspects of the ACP conversation, Prerequisites of ACP	75% of the criteria met
Livingston et al. United Kingdom, 2010	A qualitative design using focus group and individual in-depth interviews	43 FCs for the focus group interviews. 46 family carers for the individual interviews. All respondents are recruited from 4 general practices, 3 memory clinics and 5 community clinics	Carers want support from other family members and healthcare professionals when making decisions. They want to receive information well timed. Relationships between stakeholders influence ACP. Remaining QoL is important when making ACP decisions	Timely initiation of ACP, Stakeholder engagement, Important aspects of the ACP conversation, Prerequisites of ACP	75% of the criteria met
Stirling et al. Australia, 2012	A qualitative design using semi-structured, Individual, in-depth interviews and focus group interviews	13 carers of PWD. 4 community nurses, 4 community support workers and 4 counsellors from memory clinics.	Providing realistic information about dementia increases empowerment and facilitates ACP. Decision aids can support ACP. Healthcare professionals provide information selectively because they think PWD and FCs cannot cope with upsetting realities.	Important aspects of the ACP conversations	25% of the criteria met
Dening et al. United Kingdom, 2013	A qualitative design using interviews	6 PWD, 5 FCs, and 5 dyads of people with dementia and their carers from A memory service	ACP decisions have to be taken with all stakeholders. Wishes of people with dementia and their carers might differ. Information, independence and control are main themes in dementia care.	Stakeholder engagement, Important aspects of the ACP conversation, Prerequisites of ACP	50% of the criteria met
Poppe et al. United Kingdom, 2013	A qualitative design using semi-structured, in-depth, interviews	12 PWD living at home, 8 FCs and 6 staff members from memory clinics.	PWD and FCs lack knowledge about dementia and ACP. ACP should be initiated by a well informed professional soon after the diagnosis. The outcome of ACP should be well documented and available for all health service providers. A decision aid can support ACP	Timely initiation of ACP, Stakeholder engagement, Important aspects of the ACP conversation, Prerequisites of ACP	50% of the criteria met
Robinson et al. United Kingdom, 2013	A qualitative design using focus group and individual interviews	5 palliative care specialists, 10 general practitioners, 17 community nurses, 10 old-age psychiatrists, 22 mental health nurses, 6 social workers, 15 members of the ambulance services, 3 solicitors and 7 members of the voluntary sector	For the implementation of ACP concerns where expressed about the timing and initiation, the possibility to deliver the patients choice, the financial and legal aspects and the different forms of documentation.	Timely initiation of ACP, Stakeholder engagement, Important aspects of the ACP conversation, Prerequisites of ACP	50% of the criteria met
Dickinson et al. United Kingdom, 2013	A qualitative design using in-depth interviews	17 PWD and 29 FCs from local older peoples services	People with dementia undertake action for practical, financial and personal planning but have difficulties making plans for future healthcare. Barriers are: lack of awareness and knowledge of ACP, the right timing and constraints about choice of future care options	Timely initiation of ACP, Stakeholder engagement, Important aspects of the ACP conversation, Prerequisites of ACP	75% of the criteria met
Horton-Deutch et al. United States, 2007	A qualitative design using semi structured interviews	31 PWD and their FCs from a outpatient Alzheimer clinic	PWD want to make decisions with important others. PWD’s pursuit of a normal level of function influences their decision making. The decisions made are not stable over time and FCs make different decisions compared to care receivers.	Stakeholder engagement, Important aspects of the ACP conversation	50% of the criteria met
Hirschmann et al. United States, 2008	A qualitative design, using semi-structured in-depth interviews	30 PWD and their FCs. 8 of these PWD lived at home, 3 used an assisted living facility and 19 lived in a long term facility	ACP discussions should be proactive and start early. Healthcare professionals should be educated to avoid a late start. Lawyers, financial workers can play a role in decision making.	Timely initiation of ACP, Engagement of all stakeholders. Important aspects of the ACP conversation, Prerequisites of ACP	50% of the criteria met

ACP: advance care planning; PWD: people with dementia; FC: family carer

* Mixed Method Appraisal Tool

**Table 5:** Description of the selected quantitative studies.

Author	Design	Participants and settings	Main Findings	Themes	MMAT*
Hamann et al. Germany, 2011	A cross sectional survey	100 PWD, 99 FCs and their referring 93 physicians	MMSE correlates negatively with the understanding (*r* = -0.44) and reasoning (*r* = -0.27) sections of the MacCAT-T. PWD who are confident about their decisional capacities want to stay longer involved in the decision making (P = .02). There is no significant correlation between PWD’s, their relatives’ (*r* = 0.05) or their physicians’ (*r* = 0.28) confidence in the decisional making capacities of PWD. The overall estimates of FCs en physicians of the decisional preferences of PWD by is poor (Kendall’s tau, (b) rel-pat = 0.24, Kendall’s tau (b) doc-pat = 0.07)	Timely initiation of ACP, Stakeholder engagement, Important aspects of the ACP conversation	50% of the criteria met
Tay et al. Singapore, 2015	A cross sectional design. A set of standard (clinical) evaluations were administered face to face	98 PWD from a tertiary hospital in Singapore	PWD scored higher on the FAB (t = -3.65, P < .0001) when they make ACP plans or intended to do so. PWD who do not feel the urge to make future plans were less willing to engage in ACP than PWD who used more active coping strategies (t = 2.83, p = .006). PWD who intended or already made future plans had less negative attitudes towards ACP (*t* = 2.47, *p* = 0,015)	Timely initiation of ACP, Stakeholder engagement, Prerequisites of ACP	50% of the criteria met
van der Steen et al. the Netherlands, 2016	A cross sectional survey	133 GPs from Northern Ireland and 188 elderly care physicians from the Netherlands	39.8% of the GPs agreed that ACP should start at diagnosis and 45.9% strongly or moderately disagreed	Timely initiation of ACP	75% of the criteria met
Brazil et al. United Kingdom, 2015	A cross sectional survey	133 GPs from Northern Ireland	GPs moderately (45.5%) or strongly (23.5%) agree that early discussions facilitated decision making. 82.7% of the GPs agree that the GP should take the initiative for ACP. 56.4% of the GPs fear that taking the initiative increases PWD’s and the family’s anxiety. 96.3% of the GPs find including the patient and family caregiver in ACP as partners has to be a clinical practice goal.79% of the GPs agreed that PWD and their families should be informed about commonly occurring health problems in dementia. 60% of the GPs disagreed that informing PWD and their families about dementia not needed because families will witness the cognitive decline later which is sufficient	Timely initiation of ACP, Stakeholders engagement. Important aspects of the ACP conversation	50% of the criteria met
Karlawish et al. United States, 2005	A cross sectional design using semi-structured interviews, questionnaires and clinical evaluations	48 PWD and 102 FCs from a Alzheimer’s Disease Centre	PWD were labelled by psychiatrists as non-competent for medical decision making (Sn < 52%; Sp > 79%) when MMSE scores were < 19	Timely initiation of ACP	75% of the criteria met
Karel et al. United States, 2010	A mixed method study using cognitive, psychiatric capacity assessments alongside semi-structured, individual, interviews	20 PWD, 20 patients with schizophrenia and 19 cognitively healthy elderly from an outpatients clinic	PWD prefer collaborative decision making with their doctor and family. When they rate their collaboration preferences on a scale from 1 to 4, PWD prefer joined decision making with their doctor (mean 2.02) and their family (mean 1.55). For PWD it is more easy to justify their choices in terms of valued activities and relationships	Stakeholder engagement	50% of the criteria met

* Mixed Method Appraisal Tool

ACP: advance care planning; PWD: people with dementia; FC: family carer

The overall quality of the papers was moderate, with MMAT ratings of 75% (5 papers), 50% (10 papers), and 25% (1 paper) (Tables 4 and 5). The qualitative papers often lacked a description of the relation between findings and the setting of the collected data. Some papers did not clearly describe the influence of the relation between the researcher and the participants. Several quantitative papers used an inappropriate sampling procedure or had a response rate below 60%.

Analysis resulted in the following four themes related to barriers and facilitators: 1. Timely initiation of ACP; 2. Stakeholder engagement; 3. Important aspects of the ACP conversation; 4. Prerequisites for ACP.

### 1. Timely initiation of ACP

#### Facilitators of ACP addressed in qualitative research

People with dementia, their family carers, and GPs all noted that an early start facilitates ACP [32–36]. Cognitive decline was frequently given as a reason [32–35, 37, 38]. According to people with dementia and family carers, GPs should therefore timely initiate ACP [32, 37]. They also indicated that diagnostic disclosure, high impact events like a hospital admission, and ACP itself stimulated them to think about future care [32, 35, 36].

#### Barriers of ACP addressed in qualitative research

People with dementia, family carers, and GPs all referred to having difficulties with determining an optimal timing for ACP [33, 34, 37, 38].

“*The trouble with dementia is it can take a long time*, *it can take a short time*. *So I don’t know what’s the best time to do it*, *but personally I’d rather do it while I still have my wits about me*”

*(carer*, *wife)* [34].

Some family carers mentioned that people with dementia are in denial about their dementia diagnosis [35, 36] or about any possible future problems, and therefore are unwilling to participate in ACP [35]. A number of family carers and GPs stated that stress or fear caused by ACP was a reason for them not to discuss future preferences [34, 37]. The uncertainty about who should take the initiative for ACP was also mentioned as a barrier [33, 37, 38].

#### Facilitators of ACP addressed in quantitative research

The importance of early ACP initiation was noted in Brazil’s survey among GPs in Northern Ireland [39]. Here, most GPs moderately (45.5%) or strongly (23.5%) agreed that early initiation facilitated later decision making. Almost 83% of these GPs also strongly or moderately agreed that the GP should take the initiative to start ACP [39]. Van der Steen et al. reported that 92% of Dutch GPs agreed that the GP should take the initiative for ACP [40].

The importance of an early start of ACP because of the cognitive decline was addressed in several studies. In their study on participation in medical and social aspects of decision making, Hamann et al. showed that Mini Mental State Examination (MMSE) scores correlated positively with the understanding (*r* = 0.44) and reasoning (*r* = 0.27) capacities of German people with dementia [41]. Karlawish’s study on the ability of people with dementia from a memory clinic to decide on starting dementia medication, showed that those with MMSE scores below 19 were often unable to make these decisions (Sn < 52%; Sp > 79%) [42]. People with dementia from a tertiary hospital in Singapore involved in ACP scored higher on the Frontal Assessment Battery (FAB) for frontal lobe functioning than those not involved (t = -3.65, P < .0001) [43].

#### Barriers addressed in quantitative research

The difficulty of the timing of ACP was reflected in Brazil’s survey among Irish GPs: almost 40% strongly or moderately agreed that ACP should start at diagnosis, whereas 46% strongly or moderately disagreed with this statement [40]. Van der Steen et al. note that 60% of Dutch GPs wanted ACP to start at diagnosis, but 25% did not [40]. According to Tay & Davison, people with dementia who did not feel the urge to make future plans, were less willing to engage in ACP compared to those who used active coping strategies (t = 2.83, p = .006) [43]. Brazil et al. reported that 56% of the participating GPs indicated they feared that initiating ACP would unnecessarily increase the family carer’s anxiety [39].

### 2. Stakeholder engagement

#### Facilitators addressed in qualitative research

In interviews, people with dementia and family carers noted that ACP should take place with all stakeholders because of their involvement in the decision-making process. Several papers stated that regarding advance directives like living wills or lasting power of attorney, experts from outside the medical profession like lawyers or financial advisers may also need to participate [34, 36, 44, 45].

“*Resuscitation was the biggest decision… I consulted with my children and my wife’s sisters and they were all in agreement… she has gone through enough*.”

*(husband*) [36].

According to GPs, a good relation between them, the people with dementia, and family carers eased ACP; when the relationship is good, people with dementia and family carers would be more open about discussing ACP [32]. People with dementia also mentioned that if they were no longer capable of making decisions themselves, they would trust their family carers to do this for them and therefore wanted them involved. Family carers stated that they were able to fulfil this role [34, 36].

#### Barriers of ACP addressed in qualitative research

Four barriers to stakeholder engagement were mentioned. According to some family carers, a poor relation between stakeholders hampers ACP. Several family carers also stated that ACP is hindered by limited assessment of the decisional capacity of people with dementia, and because taking responsibility for ACP is difficult [36, 38]. One study mentioned that people with dementia’s unawareness of the dementia diagnosis also limits their engagement [37].

#### Facilitators of ACP addressed in quantitative research

Brazil et al. reported that 96% of the participating Irish GPs found that including people with dementia and family carers in ACP should be a goal of clinical practice [39]. People with dementia from an American outpatient clinic who were asked to rate their collaboration preferences on a scale from 1 (I want to make the decision myself) to 4 (I want my doctor or family to make the decision), preferred shared decision-making with their doctor (mean 2.02) and their family (mean 1.55) [46]. This study also showed that when ACP focused on the consequences of medical decisions and on the values of people with dementia instead of on complex treatment scenarios, people with dementia could participate longer [46]. In addition, a survey among people with dementia or with mild cognitive impairment showed that confidence in their capacity to make medical decisions was an important factor in their willingness to be engaged in ACP. Those who were confident about their decision-making capacity wanted to stay involved longer (P = .02) as opposed to those lacking confidence [41].

#### Barriers addressed in quantitative research

A survey among people with dementia or mild cognitive impairment, their relatives and physicians, showed that people with dementia were more confident about their decisional capacities compared to their relatives or physicians. There was no significant correlation between people with dementia’s confidence and their relatives (*r* = 0.05), between people with dementia and their physicians (*r* = 0.17) or between relatives and physicians (*r* = 0.28) regarding people with dementia’s medical decision-making capacities [41]. Relatives gave better estimates of the decisional preferences than physicians, but their overall estimation was poor (Kendall’s tau, (b) rel-pat = 0.24, Kendall’s tau (b) doc-pat = 0.07) [41].

### 3. Key aspects of the ACP conversations

#### Facilitators addressed in qualitative research

With respect to setting the goals they would like to achieve with ACP, people with dementia and family carers wanted to discuss a normal level of functioning and maintaining QoL [36, 44]. In addition, people with dementia, family carers and GPs stated that financial matters and the power of attorney needed to be discussed [34–36, 38]. Family caregivers and healthcare professionals added that they felt that unwanted and burdensome interventions like hospital admissions took place if these preferences remained unknown [37]. Family carers’ earlier experiences with ACP therefore stimulated the decision-making process [36]. Dickinson et al. showed that when goals are discussed, people with dementia and their family carers preferred informal discussions instead of written documents [34]. The use of decision aids providing information and structure appeared to contribute to decision-making during ACP [32, 47]. When ACP had taken place, documentation of preferences (for example in the medical file or a lasting power of attorney) was found essential, as it would make the preferences available to all stakeholders [32, 34, 35, 38].

“*So she needed to make a decision whether she would be fed by a percutaneous endoscopic gastrostomy at some point, and by the time that was a reality, the family were left to make that decision for her. And she had said, anecdotally, that she wanted the least intervention possible, but then nothing was documented … I suppose nobody took ownership or leadership of that process at all, and everyone was floundering a bit with it (social worker)*”

[38].

Family carers wanted realistic information during ACP because this increased their empowerment [37]. They also felt that GPs should ask people with dementia directly about their preferences [37].

#### Barriers addressed in qualitative research

Several studies showed that family carers and people with dementia felt they were insufficiently informed about dementia, its consequences, and care and treatment options [32–34, 36, 37].

“*Patients are often sent home with a diagnosis. They know what’s going on, but they didn’t get very specific information from the specialists. They wonder, ‘‘What will happen to me? Is there really nothing they can do for me?*”

*(Male GP)* [37].

In one study, some family carers stated that GPs selectively provided information because, if too much information was given, people with dementia and family would not be able to process this [47]. In two studies, family carers mentioned that they lacked knowledge about the purpose of ACP or that they were unaware of its existence [34, 45].

People with dementia, family carers, and GPs were all concerned that preferences for future care could not be met because of restrictions within the healthcare system [34, 36, 38, 45]. In addition, GPs stated that when people with dementia or their family carers wanted to discuss financial matters and the power of attorney, the costs for actually settling these matters were considered to be too high [38].

In two studies, people with dementia, family carers, and GPs stated that wishes were not always registered in the patient’s medical file or other formal documentation. The uncertain future and feelings of guilt or disloyalty made them reluctant to do so [34, 37].

#### Facilitators addressed in quantitative research

People with dementia emphasized they themselves want and are able to decide on social subjects within ACP. When people with dementia were asked who should have the greatest say on different subjects, (answers ranked from 1: this person should have the greatest say; to 3: this person should have the least say), people with dementia reported wanting to make their own social decisions e.g. about housing (mean rank 1.28; SD 0,6) or driving (mean rank 1.39; SD 0,63). With regard to drug related decisions, however, people with dementia wanted the physician to have the greatest say (mean rank 1.51; SD 0,7) [41].

In Brazil et al.’s study, the importance of informing people with dementia about dementia was stressed. Of all participants, 97% agreed with the statement: ‘people with dementia and their families should be informed about commonly occurring health problems that might be expected in severe dementia’ [39]. Fifty-one percent of the GPs in this study also agreed that, when dealing with dementia, documenting preferences in an advance directive was essential [39].

### 4. Prerequisites for ACP

#### Facilitators addressed in qualitative research

GPs stated in interviews that they need sufficient knowledge about the dementia disease process and its life-limiting character, and that they need training to develop the skills to discuss difficult subjects and manage conflicts [32, 37]. Some GPs added that positive previous experiences with people with dementia made them more willing to discuss ACP in the future [37].

People with dementia and family carers noted that after having had ACP consultations, they felt relieved and were more confident that their future wishes would be respected [32]. They added that ACP discussions should be repeated to enable a review of decisions and/or documentation made [32, 34, 44]. Horton Deutch et al’s finding that half of the people with dementia who were asked to make a healthcare decision based on a vignette changed their initial preferences after four weeks, supports this view [44].

#### Barriers addressed in qualitative research

In several studies, part of the GPs, family carers, and people with dementia expressed negative attitudes towards ACP because of the unpredictable nature of the disease progression. This made them question the feasibility and added value of ACP, and therefore made them unwilling to discuss future care preferences [32, 34, 38, 45].

“*You don’t know what changes will happen, when they will happen… that’s why it [ACP] is very difficult to define*.”

*(Carer)* [45]

Some people with dementia and family carers added that ‘living one day at a time’ resulted in negative attitudes towards ACP, and some people with dementia found discussing the future dispiriting [32, 34–36, 45]. Family carers also stated that the personality of people with dementia might impede ACP because, in general, they did not want to talk about difficult subjects [32, 34–36, 45].

A number of GPs felt that ACP was outside their professional remit [38]. In addition, several GPs stated that ACP was not possible because preferences might change [33, 37, 38]. They also noted barriers like their lack of knowledge regarding legal aspects in relation to ACP and the documentation of decisions in living wills, lasting power of attorney, or advance directives. This was especially true in relation to people with dementia[37, 38].

“*I get confused about the terminology about advance care and advance directive and that and one’s legal binding, and it all becomes a bit of a blur*.”

*(GP)* [38].

#### Facilitators addressed in quantitative research

According to the Perceived Barriers Scale, people with dementia who already had or intended to make future plans, had less negative attitudes towards ACP than those who did not (*t* = 2.47, *p* = 0,015) [43].

## Discussion

In this integrative review, we identified barriers and facilitators faced by GPs related to ACP for people with dementia, clustered in four themes: timely initiation of ACP; stakeholder engagement; important aspects of the ACP conversation; and prerequisites for ACP. After integrating the data, we noted slightly more facilitators than barriers. Interestingly, the selected quantitative papers mainly focused on the timely initiation of ACP and stakeholder engagement, while the qualitative papers addressed all four themes.

The most important facilitators mentioned were: an early start, when the person with dementia can still be actively involved, and the participation of all stakeholders. Diagnostic disclosure, providing information, a good relationship between all stakeholders, and discussions about social issues with a focus on people with dementia values, QoL and maintaining normal life also appeared relevant and important, as were regularly repeating ACP discussions and reviewing possible documentation, as preferences may change.

The most important barriers for ACP mentioned by all stakeholders included elements of uncertainty: the uncertainty of when to start, the uncertain future, and people with dementia’s and family carers’ lack of knowledge about dementia. GP-specific barriers were the difficulty of assessing the decisional capacity of people with dementia, the possibility that future preferences might change, and the uncertainty whether future care preferences eventually could be granted.

The reluctance to engage in ACP was also described in a systematic review by van der Steen et al. The barriers they found were mostly related to the unwillingness of people with dementia or their family carers to initiate ACP [48]. In line with our results, this review suggests that, regarding the optimal timing for ACP, the healthcare professional should initiate ACP when people with dementia and their family carers are receptive and feel the urgency to start, but before a crisis develops [48]. However, as our results show, GPs are also hesitant to initiate ACP. As stated in the review by De Vleminck et al., the dementia’s uncertain disease process is one of the causes for this hesitation [32, 34, 38, 45, 49] which may lead to a prognostic paralysis: a situation where GPs avoid discussing future care preferences [50–52]. Because GPs are used to providing reactive care, and ACP requires thinking ahead, ACP initiation becomes even more difficult [53].

Research on patients with chronic diseases shows that, even in cases where GPs want to start ACP early, patients first need time to cope with the idea of having a chronic, progressive disease [54, 55]. GPs could stimulate timely ACP initiation by regularly checking people with dementia’s readiness to start ACP, and by using cognitive or functional decline or a crisis situation as a motive [7, 48, 56–60].

Our results show that people with dementia and family carers feel insufficiently informed about dementia, which confirms the findings in the systematic reviews by Dening et al. and Gillissen et al., and in research on communication in dementia care [10, 52, 61, 62]; only informed patients are able to reflect on which options they have or which problems may arise [63]. If a person with dementia is unaware of or even denies the dementia diagnosis and therefore the possibility of future problems, the barrier to starting ACP becomes even more complex [35–37].

Initiation of ACP may also be postponed by the GPs’ and family carers’ doubts about the decisional capacities of people with dementia [37, 38]. This was also shown in the review by Gillisen et al. about ACP in long term dementia care [52]. However, the decisional capacity can differ between subjects and over time. GPs should therefore try to involve people with dementia and their family carers at every stage of the disease, and tailor ACP discussions to the specific abilities of the person with dementia in question [52, 55, 64]. A goal-oriented approach is likely to help GPs overcome this problem [65]. The use of this approach is supported by results from our review in which people with dementia emphasized the importance of maintaining normal lives, and their role in the present day where they mainly want to decide on (future) social issues [36, 44, 46]. This approach is in line with the fact that patients in general want to articulate their life’s values and use these to make decisions later on, or to have family carers decide for them [66].

ACP for people with dementia could therefore explore what is important in the present so that future care can then be planned according to these preferences [65]. Using this approach corresponds with the broad definition of ACP used in our introduction.

## Implications for practice

To improve the timely initiation of ACP, GPs need training [32, 67]. As a key message, we suggest that people with dementia participate in ACP when future care is planned in light of their goals, life values, normal daily function, and their remaining QoL [41, 46, 67]. A recently published dynamic model for shared decision making with frail elderly could be used for this purpose [65]. In this model, the patient’s near future goals are the starting point for discussing preferences for future care, and these are also regularly reviewed [65]. By using this approach, barriers regarding an uncertain future and the decisional capacities of people with dementia may become less relevant. In addition, GPs need to be aware of the consequences of dementia, including legal issues, and about the significance of informing people with dementia. This may help GPs anticipate the illness process and recognize the people with dementia’s and their carers’ need for information [37, 38, 49, 68].

Using a collaborative care model, where case managers take on GP tasks, may also stimulate a timely initiation of ACP. Research shows that case managers have regular contact with people with dementia and have sufficient communication skills to discuss difficult subjects. They are also able to coordinate care and educate people with dementia and their family carers about dementia and the legal issues concerning ACP. This approach requires regular consultations between GPs and case managers [69, 70].

The use of an ACP workbook containing information and exercises on how to communicate choices in combination with a home visit of a social worker, increased the number of ACP discussions and documentation of preferences in people with a chronic illness. This may therefore also be useful for people with dementia [71]. The Surprise Question or other tools used to identify patients in need of future care planning, may also help GPs to timely start ACP [71–73]. Financial compensation for the time spent on ACP could possibly encourage GPs to embed ACP in regular care, however there is little evidence for this [49].

## Strengths and limitations

The systematic and strong integration of qualitative and quantitative results is the main strength of this review. All the themes were covered by papers with differing methodologies, with only small differences noted. As a consequence, the themes resulting from our analysis are likely to reflect the most important barriers and facilitators for the initiation of ACP with people with dementia by GPs. As many of the selected studies were qualitative, we were able to collect additional in-depth information which may contribute to implementation of ACP solutions in primary dementia care.

One limitation of our study is that most of the articles were related to research conducted in western countries. Our results cannot therefore be generalized to non-western countries, as culture and ethnicity have a profound influence on ACP [74, 75]. Several papers included other primary care professionals in addition to GPs, therefore it was not always clear if the given data concerned the GPs. Another limitation is reflected in the quality of the papers included. None of them had a maximum MMAT rating, and the overall quality was moderate. However, no contradictory findings were reported, and most were confirmed in more than one of the included papers.

## Conclusion

Exploring people with dementia’s medical and social preferences for future care together with a focus on maintaining QoL and normal daily function may contribute to their better and longer involvement in ACP. ACP should therefore start with discussing what goals people with dementia have for the near future, which can then be used to make decisions about future care. Because of their position within the healthcare system, GPs have the opportunity to initiate ACP in primary care. Significant facilitators for this process are a timely start when cognitive decline is still mild, and the engagement of people with dementia and their family carers. To be successful, it is essential to train GPs in the skills necessary to initiate ACP discussions. This integrative review provides input for designing GP training programs, and facilitating future care planning for people with dementia in agreement with their wishes and preferences.

## Supporting information

S1 File PRISMA 2009 checklist. (PDF)
